# The Relationship Between Digital Game Addiction and Loneliness and Social Dissatisfaction in Adolescents

**DOI:** 10.7759/cureus.34604

**Published:** 2023-02-03

**Authors:** Mehmet Emin Parlak, Erdoğan Öz, Dilek Ener, Fatma Kurt, Osman Küçükkelepçe, Yaşar Kapıcı

**Affiliations:** 1 Pediatrics, Kahta State Hospital, Adıyaman, TUR; 2 Family Medicine, Adıyaman Provincial Health Directorate, Adıyaman, TUR; 3 Public Health, Kırıkkale Central Community Health Center, Kırıkkale, TUR; 4 Child and Adolescent Mental Health and Diseases, Adıyaman Training and Research Hospital, Adıyaman, TUR; 5 Public Health, Local Health Authority of Province Adiyaman, Adıyaman, TUR; 6 Psychiatry, Kahta State Hospital, Adıyaman, TUR

**Keywords:** digital addiction, adolescent, social dissatisfaction, loneliness, screen time, digital game addiction

## Abstract

Background

In this study, it was aimed to examine digital addiction, loneliness and social dissatisfaction among adolescents studying in Adıyaman, Turkey, and to determine the relationship with each other.

Methodology

Digital Game Addiction Scale for Children (DGASFC) and Loneliness and Social Dissatisfaction Questionnaire (LSDQ) were administered to 634 middle and high school students. A questionnaire form was used as a data collection tool.

Results

DGASFC scores and LSDQ scores were found to be higher in males, in high school students, in those whose parents' education level was high school or above, in those whose parents lived separately, in those with good economic status, in those who were younger, and in those who were not restricted by their families. A significant positive correlation was found between DGASFC and LSDQ scores.

Conclusions

Digital addiction should be followed closely in terms of accompanying disorders or pathologies that predispose to it. In our study, it was found that digital game addiction, loneliness and social dissatisfaction decreased with age. However, this applies separately to middle school and high school groups. Because, despite their older age, high school adolescents have been found to be more digitally dependent, lonely and socially dissatisfied than secondary school students. Contrary to the studies in the literature, the risk of digital addiction, loneliness and social dissatisfaction was found to be low in those with low economic status.

## Introduction

Digital game addiction is when the person spends time on digital games in a way that he cannot control the time. Rather than spending too much time on the game, it means seeing negative effects with it. Withdrawal symptoms occur when the game is not played. As a result of the impulse disorder, indifference towards other activities and people occurs [1].

The advancement of technology day by day and the increase in the rate of urbanization have caused digital games to take the place of traditional games. People of all ages, especially children and adolescents, show interest in digital games [2].

The restriction of activities such as education, cinema, theater, sports competitions during the COVID-19 process, and the continuation of education online have led to more use of digital technology at home. As a result, the time spent in front of the screen (mobile phone, computer, television) increased in adolescents [3]. Compared to the pre-pandemic period, students allocated more time for digital games, especially on the phone, during the pandemic period [4].

There is a significant relationship between digital game addiction and other addictions [5]. For this reason, digital addiction should be followed in order to combat other addictions, including substance abuse, in adolescents. In this study, it was aimed to examine digital addiction, loneliness and social dissatisfaction among adolescents studying in Adıyaman, Turkey, and to determine the relationship with each other.

## Materials and methods

The research is a cross-sectional and descriptive study. It was made in the city center of Adıyaman in January-February 2022. The population of the research consists of students studying in secondary and high schools in Adıyaman city center. Schools were selected by cluster sampling method. It was planned to take at least 384 people with a 95% confidence level, 0.05 margin of error, and 634 students participated in the research. The inclusion criteria of the study were being able to understand and answer the questionnaire form and living in Adıyaman. Exclusion criteria of the study were unwillingness to participate in the study and family's disapproval of the study. A questionnaire form was used as a data collection tool. Students gave assent to the study and a consent form was obtained from their parents. Ethics committee approval was obtained for the study from the Non-Interventional Clinical Research Ethics Committee of Adıyaman University, dated 26/10/2021 and numbered 2021/08-09.

Digital game addiction scale for children (DGASFC), which was developed by Hazar and Hazar in 2017, was used in our study. The scale consists of 31 questions under four items: excessive focus and conflict on playing digital games, development of tolerance during playtime and value attributed to play, postponing individual and social tasks/homework, and psychological, physiological reflection of deprivation and plunge into the game. Adolescents chose the most suitable one out of four options such as "strongly disagree", "disagree", "agree", "strongly agree" [6]. In addition, the Turkish version of the Loneliness and Social Dissatisfaction Questionnaire (LSDQ) developed by Asher and Wheeler in 1985, consisting of 23 questions, was used in our study. Adolescents chose the appropriate one out of three options as "yes", "no", and "sometimes" among the questions asked [7].

Statistical analysis

Analyses were evaluated in 22 package programs of SPSS (Statistical Package for Social Sciences) version 26.0 (IBM Corp., Armonk, NY). In the study, descriptive data were shown as n and % values in categorical data, and mean ± standard deviation (mean ± SD) and median interquartile range (25-75 percentile values) in continuous data. Conformity of continuous variables to normal distribution was evaluated by the Kolmogorov-Smirnov test. The Mann-Whitney U test was used to compare paired groups, and the Kruskal-Wallis test was used to compare more than two variables. The Spearman correlation test was used to examine the relationship between continuous variables. Linear Regression analysis was applied to determine the predictors of Digital Game Addiction Scale for Children (DGASFC) and Loneliness and Social Dissatisfaction Questionnaire (LSDQ). While creating the model, the Enter method was used, and those with a significant relationship in the correlation test were included in the model. The statistical significance level in the analyses was accepted as p<0.05.

## Results

A total of 634 adolescents with a mean age of 15.1±1.2 (min = 13 - max = 18) were included in the study. A total of 314 (49.5%) of the adolescents are boys and 320 (50.5%) are girls; 6.8% of the students study in secondary school and 93.2% in high school. The family type of 68.1% of the adolescents is nuclear family, 26.3% of them are extended families and 5.5% of them live separately from their parents. A total of 80% of the students live with their parents, 10.9% with their parent, and 9.1% with their dormitory/housemates. A total of 21.9% of the students have their own computer, 40.5% have their own mobile phone, 37.4% have their own tablet and 57.7% have their own internet connection (Table 1).

**Table 1:** All characteristics of the adolescents included in the study DGASFC: Digital Game Addiction Scale for Children; LSDQ: Loneliness and Social Dissatisfaction Questionnaire; IQR: Interquartile ranges.

		Number	%
Age, Median (IQR)		15 (14-16)	
Gender	Male	314	49.5
	Female	320	50.5
School	Middle School	43	6.8
	High School	591	93.2
Mother education status	Middle School or below	447	70.5
	High School or above	187	29.5
Father education status	Middle School or below	309	48.7
	High School or above	325	51.3
Economical situation	Low	230	36.3
	Moderate	299	47.2
	High	105	16.6
Family type	Nuclear family	432	68.1
	Extended family	167	26.3
	Parents separated	35	5.5
Number of siblings	1	50	7.9
	2	36	5.7
	3	121	19.1
	4 or above	427	67.4
Adolescents living with	Mother and father	507	80.0
	Mother or father	69	10.9
	Dorm/housemates	58	9.1
Game play time	≤1 hour	165	26.0
	2-3 hours	112	17.7
	4-5 hours	122	19.2
	6-7 hours	113	17.8
	≥8 hours	122	19.2
Boundary status of the family	Yes	409	64.5
	No	225	35.5
Own computer status	Yes	139	21.9
	No	495	78.1
Own cell phone status	Yes	257	40.5
	No	377	59.5
Own tablet status	Yes	237	37.4
	No	397	62.6
Own internet connection status	Yes	366	57.7
	No	268	42.3
Scale scores		Median (IQR)	
Hyperfocus and Conflict towards Digital Gaming		12 (8-15)	
Tolerance Development in Playtime and Value Added to Play		14 (9-17)	
Postponing Individual and Social Tasks/Homework		9 (6.0-12)	
The Psychological Physiological Reflection of Deprivation and Plumbing		6 (4-8)	
DGASFC		41 (30-50)	
LSDQ		26 (22-31)	

Comparison of scale total scores according to various parameters is shown in Table 2. The boys' DGASFC (p=0.013) and LSDQ (p=0.034) scores were higher than the girls' scores. DGASFC (p=0.009) and LSDQ (p=0.003) scores of high school students were found to be significantly higher than those of secondary school students. The DGASFC score of those whose mother and father education level is high school or higher was found to be significantly higher than the score of those whose mother and father education level is secondary school or below (p<0.001). The LSDQ score of those whose mother's education level (p=0.042) and father's education level (p<0.001) were high school and above was found to be significantly higher than those who graduated from secondary school or below. There was a significant difference between economic status in terms of DGASFC (p=0.003) and LSDQ (p=0.014) scores. Those with lower economic status had lower scores.

**Table 2:** Comparison of scale total scores according to various parameters DGASFC: Digital Game Addiction Scale for Children; LSDQ: Loneliness and Social Dissatisfaction Questionnaire; IQR: Interquartile ranges. *Mann-Whitney U test was used for two-category comparisons and the Kruskal-Wallis test was used for three-category comparisons. The statistical significance level in the analyses was accepted as p<0.05.

		DGASFC		LSDQ	
		Median (IQR)	p^*^	Median (IQR)	p^*^
Gender	Male	43 (32-51)	0.013	27 (22-32)	0.034
	Female	38 (28-50)		26 (22-29)	
School	Middle School	27 (24-50)	0.009	26 (22-30)	0.003
	High School	41 (31-51)		28 (25-33)	
Mother education status	Middle School or below	39 (29-50)	<0.001	26 (22-30)	0.042
	High School or above	45 (33-53)		27 (23-32)	
Father education status	Middle School or below	37 (27-49)	<0.001	26 (22-29)	<0.001
	High School or above	43 (32-53)		27 (23-32)	
Economical situation	Low	38.5 (28-50)	0.003	26 (22-30)	0.014
	Moderate	41 (30-52)		27 (22-31)	
	High	44 (35-52)		27 (23-32)	
Family type	Nuclear family	41 (30-50)	<0.001	26 (22-30)	<0.001
	Extended family	39 (31-52)		26 (22-31)	
	Parents separated	70 (35-90)		40 (27-40)	
Adolescents living with	Mother and father	39 (30-49)	<0.001	26 (22-29)	<0.001
	Mother or father	50 (32-86)		29 (22-40)	
	Dorm/housemates	45.5 (34-89)		29 (26-40)	
Boundary status of the family	Yes	39 (30-50)	0.039	26 (22-29)	<0.001
	No	44 (31-53)		28 (24-32)	
Own computer status	Yes	46 (35-58)	<0.001	27 (22-33)	0.046
	No	39 (30-50)		26 (22-30)	
Own cell phone status	Yes	41 (32-53)	0.028	27 (22-32)	0.030
	No	41 (30-50)		26 (22-29)	
Own tablet status	Yes	44 (33-54)	<0.001	27 (23-32)	0.002
	No	38 (28-50)		26 (22-30)	
Own internet connection status	Yes	42 (32-52)	<0.001	26 (23-31)	0.002
	No	39 (28.5-50)		26 (21-30)	

There was a significant difference between family type in terms of DGASFC (p<0.001) and LSDQ (p<0.001) scores, and this difference was due to the difference between the adolescents with separate parents and the other two groups, and the scores of those whose parents were separated were found to be higher.

There was a significant difference between the adolescents living with their parent/parents in terms of DGASFC (p<0.001) and LSDQ (p<0.001) scores, and the scores of those living with their parents were found to be lower.

The DGASFC (p=0.039) and LSDQ (p<0.001) scores of those whose family set limits were found to be significantly lower than those whose family did not. Those who had their own computer (p<0.001), those who had their own mobile phone (p=0.028), those who had their own tablet (p<0.001) and those who had their own internet connection (p<0.001) had significantly higher DGASFC scores. Likewise, those who had their own computer (p=0.046), those who had their own mobile phone (p=0.030), those who had their own tablet (p=0.002) and those who had their own internet connection (p=0.002) had significantly higher LSDQ scores.

**Figure 1:**
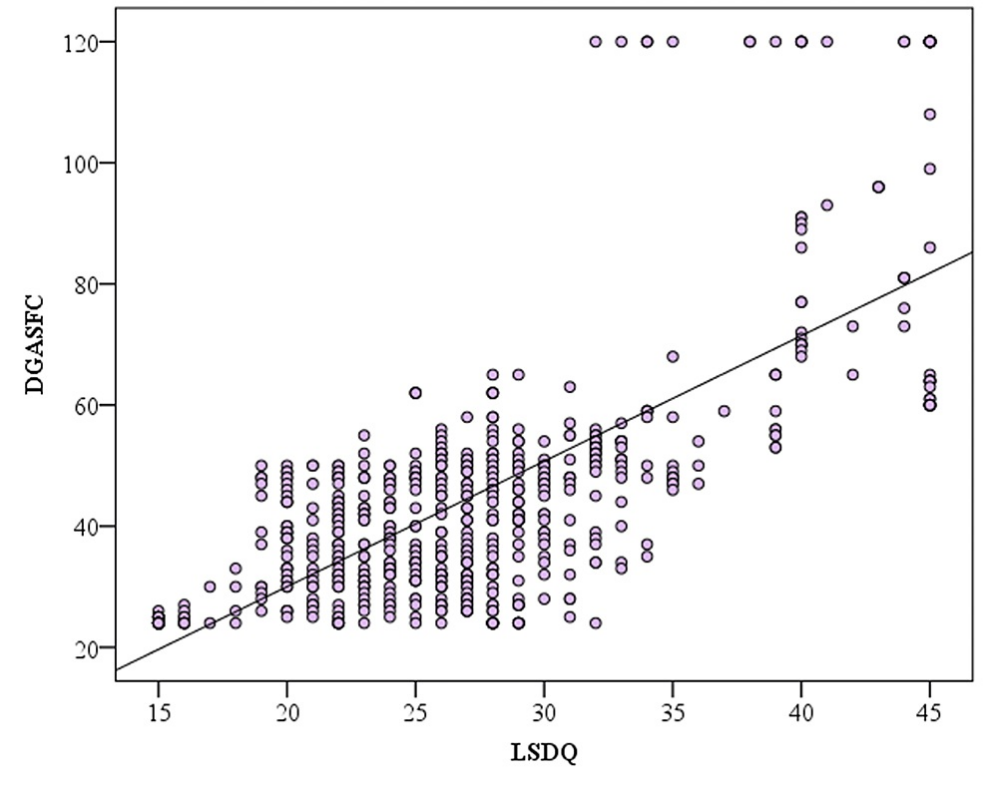
Correlation of DGASFC total score DGASFC: Digital Game Addiction Scale for Children; LSDQ: Loneliness and Social Dissatisfaction Questionnaire.

The correlation analysis between DGASFC and LSDQ and game playing time is shown in Table 3. There was a positive correlation between DGASFC total score and LSDQ and playing time. There was a significant negative correlation between DGASFC and age. There was a positive correlation between LSDQ and playing time (Figure 1). There was a significant negative correlation between LSDQ and age.

**Table 3:** Correlation of scale scores DGASFC: Digital Game Addiction Scale for Children; LSDQ: Loneliness and Social Dissatisfaction Questionnaire. Spearman correlation analysis was applied. The statistical significance level in the analyses was accepted as p<0.05.

	DGASFC		LSDQ	
	r	p	r	p
LSDQ	0.666	<0.001		
Age	-0.130	0.001	-0.094	0.018
Game play time	0.522	<0.001	0.355	<0.001

According to the multiple linear regression analysis, LSDQ score (β=1.801, p<0.001), playing time (β=3.022, p<0.001) and age (β=-1.290, p=0.008) predicted the DGASFC total score. LSDQ total score was predicted by DGASFC total score (β=0.239, p<0.001) and playing time (β=0.483, p=0.002) (Table 4).

**Table 4:** Linear regression analysis of factors associated with DGASFC and LSDQ DGASFC: Digital Game Addiction Scale for Children; LSDQ: Loneliness and Social Dissatisfaction Questionnaire; SE: Standard error. Linear regression analysis was applied. The statistical significance level in the analyses was accepted as p<0.05.

	β	SE	Standard β	t	p
DGASFC (R^2^=0.567; F=277.217; p<0.001)					
LSDQ	1.801	0.083	0.633	21.796	<0.001
Game play time	3.022	0.415	0.210	7.280	<0.001
Age	-1.290	0.483	-0.071	-2.671	0.008
LSDQ (R^2^=0.536; F=244.264; p<0.001)					
DGASFC	0.239	0.011	0.679	21.796	<0.001
Game play time	0.483	0.156	0.096	3.096	0.002
Age	-0.206	0.177	-0.032	-1.168	0.243

## Discussion

Applications to child and adolescent psychiatry clinics with digital addiction complaints are increasing day by day [1]. The COVID-19 pandemic has caused this increase to become evident [4,5,8]. Digital game disorders are more common in people with high levels of depression, anxiety, and stress [8]. For this reason, people with digital addiction may have an underlying psychiatric illness. In addition, an increase in activity was detected in the regions of their brains associated with reward, addiction, desire and emotion, similar to that of other addicts [5].

In our study, boys' DGASFC (p=0.013) score was found to be higher than that of girls. This finding is consistent with Söyöz Semerci and Balcı's study among high school students in 2020, Hazar et al.'s study among secondary school students in 2020, Aydın Özgür's study among secondary school students in 2020, and Ekinci et al.'s study among secondary school students in 2019 [9-12]. However, in the study conducted by Kılıç in 2020 among high school students, digital addiction was found to be higher among female students [13].

In our study, the boys' LSDQ (p=0.034) score was found to be higher than the girls' score. In the study of Ekinci et al. and Kılıç, no correlation was found between gender, loneliness and social relations [12,13]. In a review by Cole et al. in 2021, it was stated that there was no gender difference in the measurement of loneliness in children and adolescents [14]. In a meta-analysis conducted by Maes et al. in 2019, it was reported that male adolescents in some studies and girls in some studies suffer from loneliness more [15].

In our study, the DGASFC (p=0.009) score of high school students was found to be significantly higher than the score of those studying at secondary school. A significant negative correlation was found between DGASFC and age. In other words, the lower the age of middle school and high school children, the more digital addiction is seen.

Hazar et al. and Ekinci et al. stated that digital addiction increases with age in secondary school students [10,12]. In a study conducted by Müller et al. in 2015, they found that digital addiction increases with age in adolescents [16]. However, Frölich et al. stated that digital addiction in adolescents does not change with age [17].

In our study, the LSDQ (p=0.003) score of high school students was found to be significantly higher than the score of those studying at secondary school. There was a significant negative correlation between LSDQ and age. That is, the lower the age of middle school and high school children, the more loneliness and social dissatisfaction are seen. The fact that the risk of both digital addiction and loneliness and social dissatisfaction is higher in high school where older children attend shows that factors other than age are more prominent in high school. Rather than comparing middle school and high school students with each other, it would be a more accurate approach to evaluate both groups within their own group.

Ekinci et al. stated that the loneliness levels of secondary school students did not change with age [12]. Kılıç stated that age does not make a difference in the levels of loneliness and social relations in high school children [13].

In our study, the DGASFC score of those whose mother and father education level is high school or higher was found to be significantly higher than the score of those whose mother and father education level is secondary school or below (p<0.001). In the study conducted by Hazar et al. in 2020 among secondary school students, it was stated that the education level of the parents did not affect the DGASFC score [10]. In a study conducted by Aydın Özgür in 2020 among secondary school students, it was determined that digital addiction was higher in those with a low educational level of the mother, but the education level of the father did not affect the digital addiction of the child [11].

In our study, the LSDQ score of those whose mother's education level (p=0.042) and father's education level (p<0.001) were high school and above was found to be significantly higher than those who graduated from secondary school or below. In the study conducted by Kılıç in 2020, no difference was found in high school students in terms of education level of mothers and fathers and loneliness and social relations [13].

Contrary to the studies in the literature, the DGASFC (p=0.003) and LSDQ (p=0.014) scores of those with low economic status were found to be statistically lower in our study. In a study conducted by Aydın Özgür in 2020 among secondary school students, the risk of digital game addiction was found to be higher for students with low household income [11]. In the studies of Kılıç and Faidah et al., it was stated that digital addiction is higher in adolescents from low socio-economic backgrounds [13,18].

In our study, both DGASFC and LSDQ scores were found to be high in those who had their own computer, phone, tablet, and internet. For this reason, digital addiction, loneliness and social dissatisfaction may be seen less in those who are in a low economic situation where they cannot afford a private computer, phone, tablet or internet. In our study, there was a significant difference between the adolescents living together in terms of DGASFC (p<0.001) and LSDQ (p<0.001) scores. This difference was due to the difference between those living with their parents and the other two groups, and the scores of those living with their parents were found to be lower. Similar to our results, Faidah et al. stated that digital addiction is high in adolescents whose parents live separately [18].

There are many studies in the literature showing that there is a direct relationship between playing time and digital addiction [13,19]. In our study, a positive and significant correlation was found between DGASFC total score and playing time. In our study, a significant positive correlation was found between DGASFC total score and LSDQ. Kılıç, in his study of high school children, stated that digital game addiction and social and emotional loneliness levels have increased [13]. In a study conducted by Savci and Aysan in 2017, they stated that digital addiction predicts social connectedness by 25%, and as a result, loneliness and social dissatisfaction develop in adolescents [20].

In our study, a positive correlation was found between LSDQ and playing time. Similarly, in their study conducted in 2019, Hayırcı and Sarı found that the time spent on the phone to access the Internet and play digital games was associated with loneliness in high school students [21].

In our study, the risk of digital game addiction, loneliness and social dissatisfaction was lower in children who were restricted by their families. However, in a study by Schneider et al. in 2017, it was stated that setting limits for digital games might work if there is a consensus between parents and adolescents [22].

The study was conducted in Adıyaman, a province of Southeastern Anatolia Region, which has the lowest socioeconomic level in Turkey. For this reason, compared to big cities, opportunities for young people in this region are very limited. This situation may have caused the youth to become more individualized and prone to addiction. In addition, Adıyaman has a high rate of young population as the fertility rate is high. The high rate of young population in the region can be considered as a limitation in the generalization of the result of the study.

## Conclusions

Our study showed that there is a significant positive correlation between digital addiction and loneliness and social dissatisfaction in adolescents. For this reason, digital addiction should be closely monitored in terms of existing comorbidities or pathologies that it predisposes to. In addition, it was found that digital game addiction and loneliness and social dissatisfaction decreased with age. However, this applies separately to middle school and high school groups. Because despite their older age, high school adolescents are more likely to have digital addiction, loneliness and social dissatisfaction than secondary school students. In short, the findings of early adolescence and late adolescence should be compared separately within their own groups, due to the change of possible interests and problems of children. In our study, it was found that digital addiction, loneliness and social dissatisfaction differ according to gender, age, education of parents, separation of mother and father, and economic status of the family. In our study, contrary to the studies in the literature, the risk of digital addiction, loneliness and social dissatisfaction was found to be low in those with low economic status. It has been determined that the risk of digital game addiction, loneliness and social dissatisfaction decreases in those who have been restricted for digital games by their families.
